# Snapping Knee Caused by Medial Meniscal Cyst

**DOI:** 10.1155/2014/151580

**Published:** 2014-04-13

**Authors:** Tsuyoshi Ohishi, Daisuke Suzuki, Kazufumi Yamamoto, Tomohiro Banno, Hiroki Ushirozako, Yoichi Koide, Yukihiro Matsuyama

**Affiliations:** ^1^Department of Orthopaedic Surgery, Enshu Hospital, 1-1-1 Chuo, Naka-ku, Hamamatsu, Shizuoka 430-0929, Japan; ^2^Koide Clinic, Kosai, Shizuoka 431-0442, Japan; ^3^Department of Orthopaedic Surgery, Hamamatsu University School of Medicine, Hamamatsu, Shizuoka 431-3192, Japan

## Abstract

Snapping phenomenon around the medial aspect of the knee is rare. We present this case of snapping knee caused by the sartorius muscle over a large medial meniscal cyst in a 66-year-old female. Magnetic resonance images demonstrated a large medial meniscal cyst with a horizontal tear of the medial meniscus. Arthroscopic cyst decompression with limited meniscectomy resulted in the disappearance of snapping, and no recurrence of the cyst was observed during a 2-year follow-up period.

## 1. Introduction

Meniscal cyst causes various symptoms depending upon its size and the site of its origin. It sometimes grows large enough to limit the patient's activities of daily living [1, 2]. Several pathologies of snapping syndrome around the medial aspect of the knee such as hamstring subluxation over the posteromedial corner of the tibia [3–5] and hamstring tendons moving back and forth over osteochondroma of the medial aspect of the proximal tibia [6] have been reported, but they are still limited in number. We present here an unusual case with snapping knee caused by the sartorius muscle over a voluminous medial meniscal cyst.

## 2. Case Report

**Figure 1:**
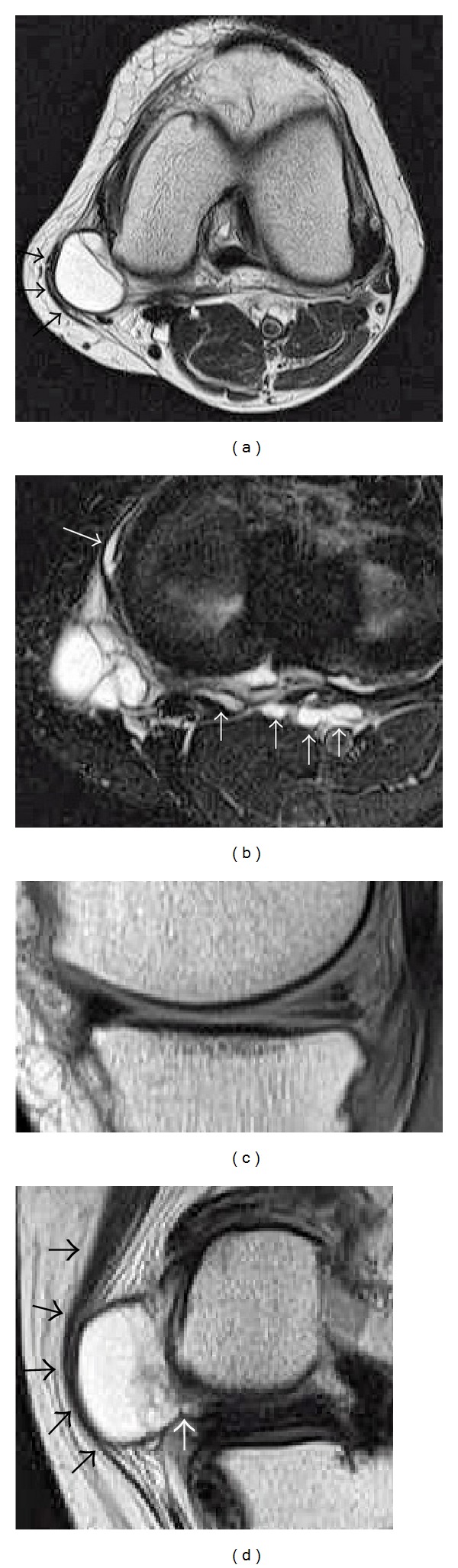
Preoperative magnetic resonance images of the left knee. (a) T2 weighted axial MRI shows a cystic mass measuring 23 mm × 20 mm detected on the posteromedial aspect of the knee. Flattened sartorius muscle (arrows) is noted just over the cyst. (b) Multiple daughter cysts expanding from the main large cyst to posterior and anterior directions were noted on STIR axial image (arrows). (c) A horizontal tear of the posterior segment of the medial meniscus was identified on sagittal proton image. (d) A communicating tract between the cyst and medial meniscus was identified (white arrow). Note that the flattened sartorius muscle is indicated by black arrows.

A 66-year-old female was referred to our hospital with a 9-month history of left knee pain. The patient recalled no trauma about the affected knee. She had felt a soft mass gradually growing around the posteromedial aspect of her left knee 3 months before her initial visit. She was 154 cm in height and body weight of 50 kg with negative findings from blood and urine examinations. Clinical examinations of the left knee revealed a palpable elastic soft mass that measured around 4 cm × 3 cm with tenderness of the posteromedial aspect of the knee. The range of motion was full but painful snapping of the hamstrings could be reproduced over the mass on active flexion and extension in an arc of 30 to 60 degrees (Video 1 in Supplementary Material available online at http://dx.doi.org/10.1155/2014/151580). No swelling, warmness, erythema, tenderness, or hydrops was found other than the medial mass about the knee. A McMurray test elicited pain over the medial aspect of the joint line. No instability was found. Plain radiography revealed grade II osteoarthritis by Kellgren-Lawrence classification in the medial compartment of the left knee. On magnetic resonance images (MRIs), a voluminous lobulated mass measured 40 mm × 23 mm × 20 mm with low intensity on T1 weighted images and high intensity on T2 weighted images was noted just under the flattened sartorius muscle on the posteromedial aspect of the knee (Figure 1(a)). Multiple small cysts extending from the major cyst to the posterior and anteromedial aspect of the knee were also noted (Figure 1(b)). A horizontal tear of the middle and posterior segment of the medial meniscus with a communicating tract to the cyst were identified (Figures 1(c) and 1(d)). A diagnosis of a medial meniscal cyst communicating with the horizontal medial meniscal tear with clinical presentation of snapping by the sartorius muscle over the cyst was made.

**Figure 2:**
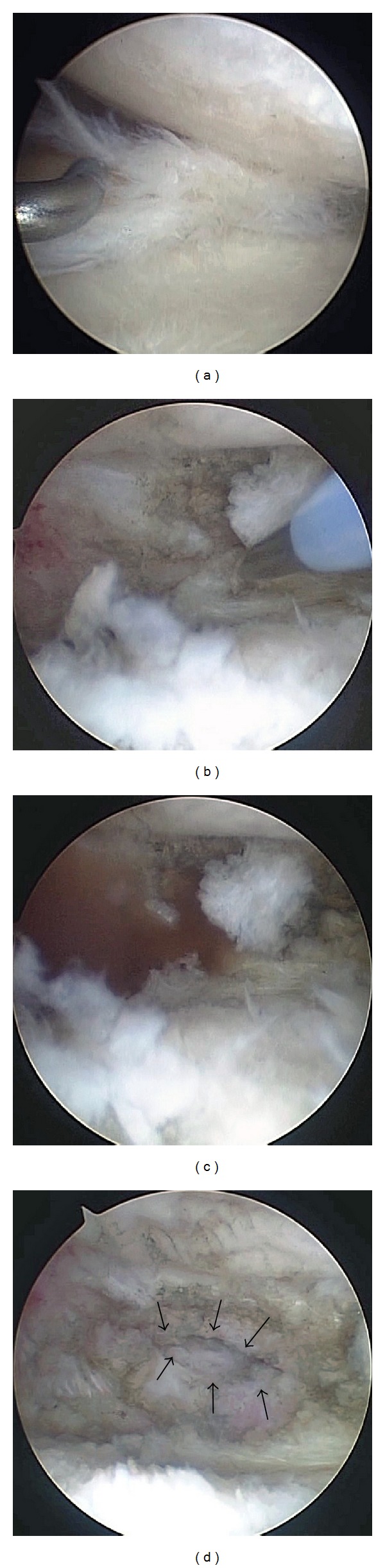
Arthroscopic views of the medial meniscus. (a) Degenerated horizontal tear of the middle and posterior segments was identified. (b) An introducer needle was inserted at the most peripheral site to the torn meniscus. (c) Massive bloody viscous fluid exploded from the small channel between the upper and lower leaf of the torn meniscus with disappearance of the medial large mass. (d) A small channel is indicated by arrows.

**Figure 3:**
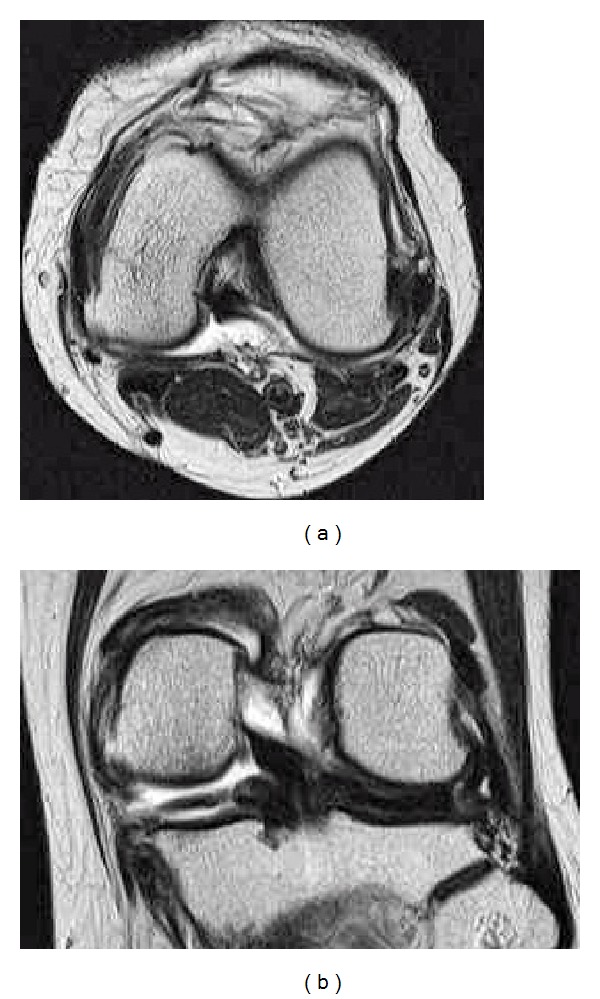
T2 weighted axial (a) and coronal (b) MR images taken 9 months postoperatively demonstrate complete disappearance of the medial meniscal cyst.

Arthroscopic surgery was performed under general anesthesia after informed consent was obtained from the patient. Anterior and posterior cruciate ligaments, cartilage in the lateral compartment, and lateral meniscus were normal. Cartilage of both the medial femoral condyle and tibial plateau had degenerated to grade II by Outerbridge classification. Extensive horizontal tear of the middle and posterior segments of the medial meniscus was identified (Figure 2(a)). Partial meniscectomy of the torn meniscus was performed until the medial capsule was exposed. Viscous reddish intracystic fluid was aspirated through the 16-gauge introducer needle inserted to the capsule between the upper and lower leaf of the horizontally torn meniscus. A small channel was created at the site where the introducer needle was inserted; creation of the small channel caused a massive amount of bloody viscous fluid to explode into the intra-articular cavity, enabling the medial large mass to disappear (Figures 2(b)–2(d)). Arthroscopic procedure for cyst decompression was provided in Video 2. Full weight bearing gait and full range of motion of the knee were allowed with an elastic bandage on the knee from 1 day postoperatively. The patient's postoperative course was uneventful. Snapping around the medial aspect of the knee disappeared completely immediately after the operation. Magnetic resonance images taken 9 months after the operation revealed all cysts including the medial large one and posterior and anteromedial multiple daughter types disappeared completely (Figures 3(a) and 3(b)). The patient felt slight pain from the medial joint space during gait but she had no difficulty with activities of daily living 2 years postoperatively.

## 3. Discussion

Frequency of meniscal cyst was 4% on MRI among 2,572 patients who were suspected to have internal derangement of the knee according to Campbell et al. [7]. Medial meniscal cysts were more common than lateral on evaluation by MRI than previously thought [7–9]. Symptoms caused by meniscal cyst varied depending upon its size and originating site. Most meniscal cysts detected by MRI presented with no symptoms, especially medial ones [7, 9, 10]. However, when a meniscal cyst expanded outside the joint, it presented as a spontaneously or intermittent palpable mass with or without pain [1, 2]. Medial meniscal cysts were more likely to extend away from their site of origin since the medial collateral ligament limited the direction of cyst expansion [2]. They tended to be larger than lateral ones since thicker fat and muscles on the medial side of the knee compared to those on the lateral side could hide the palpation of growing mass [1, 2, 8]. Therefore, a patient might visit the hospital when a meniscal cyst grows large enough to be palpated as in the case of a medial meniscal cyst.

Snapping syndromes are uncommon around the medial aspect of the knee. So far, snapping caused by subluxation of the semitendinosus and gracilis tendons over the posteromedial corner [3–5] and the movement of hamstring tendons over osteochondroma at the medial border of the proximal tibia was reported [6]. Snapping knee caused by the sartorius muscle moving back and forth over a large medial meniscal cyst during knee motion has not been reported in the literature.

In this case, the meniscal cyst derived from the posterior segment of the medial meniscus expanded just posterior to the medial collateral ligament under the sartorius muscle. The growing pressure of the cyst against the muscle flattened the sartorius muscle. Snapping was generated during the knee motion since the sartorius muscle moves posteriorly during flexion of the knee over the cyst that was fixed on the capsule, and vice versa, during extension. Multiple daughter cysts posterior and anteromedial to the tibia were identified on MRI preoperatively. Obstruction of enlargement of the cyst in the medial direction by the sartorius muscle might result in posterior and anterior expansion of the cyst.

For the treatment of meniscal cyst, limited meniscectomy with cyst decompression under arthroscopy or open cystectomy has been recommended [11–14]. Moreover, arthroscopic repair of the torn meniscus after cyst decompression has been also reported [15, 16]. In our patient, an arthroscopic partial meniscectomy with cyst decompression was chosen since the patient was older and the type of meniscal tear was a large degenerative horizontal tear. Not only medial large cysts but also posterior and anteromedial multiple cysts disappeared concomitantly as the result of this procedure. Open cyst resection could result in persistence of the posterior and anterior multiple daughter cysts. In this case, a communicating tract between the cyst and torn medial meniscus was identified on MRI; however, it was not found during arthroscopy. A communicating hole was therefore created on the medial capsule that was most peripheral to the site of the torn meniscus. Howe and Koh demonstrated excellent results in a long follow-up period by creating a small channel to the capsule adjacent to the cyst for decompression of the cyst into the joint [17]. Indeed, there may be many cases where the communicating tract is too small to identify during arthroscopic surgery. Therefore, with arthroscopic cyst decompression, if no communicating tract can be found at the original site it will be necessary to make a communicating tract between the cyst and the site of the torn meniscus.

## Supplementary Material

Video 1: Snapping phenomenon on the medial aspect of left knee is shown. Hamstring tendons move on the medial mass with a snap during active flexion and extension in an arc of 30 to 60 degree of the knee.
Video 2: An overview of the arthroscopic procedure. Degenerative horizontal tear of the posterior segment of the medial meniscus is identified by probing. After resection of the torn meniscus through the anteromedial and anterolateral portals, a cleft between upper and lower leaf of the horizontally torn meniscus is clearly seen. A 16-gauge introducer needle is inserted to the cleft. Then, bloody viscous fluid gushes out. A small channel between cyst and joint is created and enlarged by a radiofrequency device.
